# DynOVis: a web tool to study dynamic perturbations for capturing dose-over-time effects in biological networks

**DOI:** 10.1186/s12859-019-2995-y

**Published:** 2019-08-13

**Authors:** T. J. M. Kuijpers, J. E. J. Wolters, J. C. S. Kleinjans, D. G. J. Jennen

**Affiliations:** 1 Department of Toxicogenomics, GROW School for Oncology and Developmental Biology, Maastricht University, P.O. Box 616, Maastricht, 6200 MD The Netherlands; 2 Present Address: School for Mental Health and Neuroscience (MHeNS), University Eye clinic Maastricht, Maastricht University Medical Centre + (MUMC+), P.O. Box 5800, Maastricht, 6229 HX The Netherlands

**Keywords:** Dynamic network visualization, Biological knowledge integration, Network analysis

## Abstract

**Background:**

The development of high throughput sequencing techniques provides us with the possibilities to obtain large data sets, which capture the effect of dynamic perturbations on cellular processes. However, because of the dynamic nature of these processes, the analysis of the results is challenging. Therefore, there is a great need for bioinformatics tools that address this problem.

**Results:**

Here we present DynOVis, a network visualization tool that can capture dynamic dose-over-time effects in biological networks. DynOVis is an integrated work frame of R packages and JavaScript libraries and offers a force-directed graph network style, involving multiple network analysis methods such as degree threshold, but more importantly, it allows for node expression animations as well as a frame-by-frame view of the dynamic exposure. Valuable biological information can be highlighted on the nodes in the network, by the integration of various databases within DynOVis. This information includes pathway-to-gene associations from ConsensusPathDB, disease-to-gene associations from the Comparative Toxicogenomics databases, as well as Entrez gene ID, gene symbol, gene synonyms and gene type from the NCBI database.

**Conclusions:**

DynOVis could be a useful tool to analyse biological networks which have a dynamic nature. It can visualize the dynamic perturbations in biological networks and allows the user to investigate the changes over time. The integrated data from various online databases makes it easy to identify the biological relevance of nodes in the network. With DynOVis we offer a service that is easy to use and does not require any bioinformatics skills to visualize a network.

## Background

The development of high-throughput sequencing techniques allows us to obtain complex data sets, which are capable of revealing molecular responses of cellular processes [1]. For instance, changes in gene expression play a role in signal transduction mechanisms, metabolic pathways and responses to harmful events in the cell [2]. For enabling a deeper mechanistic understanding these data sets have necessitated the development of mathematical models. For example, transcriptomic data have been used to construct gene regulatory networks, which provide valuable insights in the regulatory mechanisms of differential gene expression [3–5].

However, it is well known that these changes in biological processes are not static but dynamic. Therefore, currently, high-throughput sequencing techniques are combined with time series experiments. Although this approach will increase the overall knowledge of dynamic cellular responses, temporal analysis adds another layer of complexity. Multiple tools have been designed to translate experimental results into network graphs for the purpose of studying time series data [6, 7] or dynamic perturbations, such as dose-over-time effects [8] . Currently there are a number of different tools available that focus on visualizing dynamic networks [9–11], but these do not focus on the integration of dynamic visualization with biological knowledge.

To improve upon this, we developed a tool that integrates dynamic network visualization with functional biological information. To visualize and understand the influence of time and dose on cellular responses, integration of high throughput sequencing time series data has been implemented. To enable a functional interpretation of different nodes and interactions in the established networks, information extraction methods have been developed to pull relevant information from various biological databases.

### Implementation

**Fig. 1:**
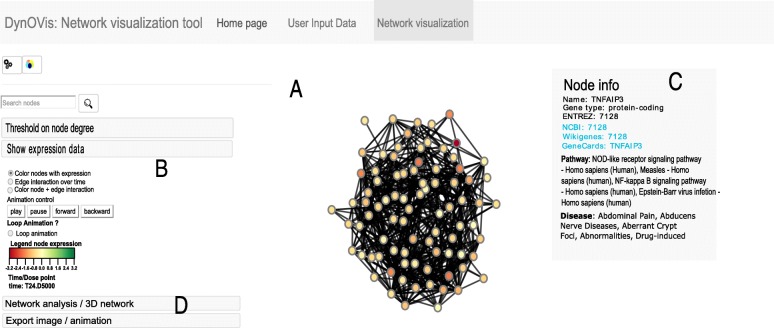
DynOVis: network visualization frame. In the centre, the network is displayed (**a**). The control panel with the threshold degree and animation controls are placed at the left (**b**) and the biological information panel for each node is shown at (**c**) after a node has been clicked. Different graph theory features have been integrated to identify important HUB nodes (**d**)

DynOVis offers an easy web-based tool for visualizing dynamic gene expression data on a biological network and is made freely available at https://bitbucket.org/mutgx/dynovis/src for downloading and running locally*.* DynOVis is an integrated work frame of R packages and JavaScript libraries, and is made freely available using the R Shiny package. R shiny is used to control the webpage and the web server, whereas the D3js JavaScript library is used to visualize the network. DynOVis is designed to guide the user through the different steps that translate a network structure into a network image. The user can decide to upload a static or dynamic network (directed or undirected) but also if they want to map biological knowledge onto the network. After the network has been constructed, the user can start analysing the network (Fig. 1).

### Initialization and creation of the network

DynOVis allows for handling different network structures, that can either be an edge list (format: A interacts with B), adjacency matrix (format: matrix of 0 and 1’s that define an interaction between nodes) or a Cytoscape file (in case previous analyses of the relevant data set has been performed using Cytoscape). We have provided a workflow that handles the processing of the network file into a network structure. Therefore, DynOVis does not require the user to specify the extension of the network file but it will automatically detect the file type and process it. The network structure is then converted into a network graph by applying the D3js force-directed graph algorithm (Fig 1a). This algorithm calculates the position of every node by applying an attractive force between each pair of connected nodes, as well as a repulsive force between the nodes. This will create a network with the least amount of overlapping nodes, which is important for studying any network. After the network has been created, the user is also free to drag and place nodes at different places and change the node positions.

### Analysing experimental data in your network

In its most general appearance, a network is a collection of nodes and the interaction between the nodes is defined by edges. These nodes may represent different entities, for instance, genes if one has built a gene regulatory network or CpG islands if one has built an epigenomic network. To investigate the relationship between changes in expression of nodes, the user may also upload a file containing expression data generated from dose, time or a combination of dose-over-time series experiments. DynOVis does not request for a specific expression format, such as intensity or log fold change and the user is free to use their own desired format.

DynOVis applies a mapping function to assign the experimental data to the corresponding node and translates the value into a colour by a red/green or red/blue linear colour scale function. To determine the domain of the colour scale, a built-in DynOVis function calculates the maximum absolute value of the experimental data is calculated and used as the left and right boundary of the scale. By using the absolute maximum value, we ensure that a dark red colour (meaning very low expression) is equal to a bright green (or blue) colour (meaning high expression).

### Analysing dynamic events on the network

DynOVis offers different options for analysing dynamic perturbations in a biological network to study the changes in expression or interactions between nodes in the network (Fig 1b). First, the user can upload a dynamic node expression file, from which DynOVis builds an animation that shows the changes in expression over time (see first example case study). Second, it is also possible to upload a dynamic edge interaction file that can be used to study changes in interactions between nodes (see second example case study). Third, both the dynamic edge and node expression file may be used to create an animation that shows how differences in node expression are associated with differences in edge interactions. This animation is to be viewed as a video, but also frame-by-frame by using the forward and backward animation control buttons. DynOVis has a built-in function that creates a 2D line graph, showing the changes in node expression if the user selects a local neighborhood of nodes. The degree of each node may change over time, which is visualized by either changing the degree of the nodes during the animation or by looking at the node degree table. This will highlight the importance of certain nodes at a given time point. Furthermore, it is possible to save the animation and use it in a presentation or on a website.

### Integrated database to the network

To increase the understanding of the different nodes in a network, it is possible to add biological knowledge to the network by making use of the internal database of DynOVis. This database is built from various online sources such as ConsensusPathDB [12], Comparative Toxicogenomics database [13] and the NCBI database [14] and allows the user to access this information without having to visit any of those websites. To get the biological information for each node, the node identifier is connected with the corresponding key identifier in the DynOVis database. The user can select during the initial stage of building the network to incorporate gene information for *Homo sapiens*, *Rattus norvegicus* or *Mus musculus*. This information includes pathway-to-gene associations from ConsensusPathDB [12], disease-to-gene associations from the Comparative Toxicogenomics databases [13], as well as Entrez gene ID, gene symbol, gene synonyms and gene type from the NCBI database [14]. Here, we have developed a function that shows a pop-up window with the biological information that only becomes visible when the user clicks on a node (Fig 1c).

### Change the node size based on their degree

Further important features in DynOVis refer to the basic network analysis options (Fig 1d). These options are derived from graph theory properties and give an overview of the most important nodes, based on degree centrality. Nodes with many connected neighbors have a high degree and are therefore called HUB nodes. It is important to identify these HUB nodes since biological networks are robust against perturbations, but disruption of pivotal nodes in general causes the system to fail [15, 16]. In DynOVis, we have split degree centrality into three components: i) total degree, ii) inner degree and iii) outer degree. This separation helps to identify a node that is regulated by many other neighbours (inner degree) or a node that regulates many nodes (outer degree). The visualization style of the nodes (i.e. the size, a property defined by D3js) can also be changed according to the different degrees, or nodes in the network can be hidden if a chosen degree is below a certain threshold.

### Study the network in three dimensions

The two-dimensional network (Fig 1a) can also be converted in a three-dimensional network. This allows the user to navigate through the network in a first-person view while at the same time playing the animation showing the dynamic perturbation. DynOVis uses a custom developed 3D force directed layout by adding an extra formula to calculate the z-position with respect to the x and y values of the 2D D3js force-directed layout by defining the following formula:

$$ z=\kern0.5em {y}_{value}\ast \kern0.5em 0.8\ast \kern0.5em \sin \kern0.5em \ast \kern0.5em \left({y}_{value}\ast \kern0.5em \pi \right)\kern0.5em +\kern0.5em {x}_{value}\kern0.5em \ast \kern0.5em 0.8\ast \kern0.5em \sin \kern0.5em \left({x}_{value}\kern0.5em \ast \kern0.5em \pi \right) $$

### Finding pathways in your network

For each gene in the network, the associated pathways are searched for in the database that is attached to DynOVis. Since one gene may be associated to multiple pathways, the number of different pathways may be very high, depending on the number of genes in the network. Therefore, DynOVis ranks the pathways based on the number of genes associated and returns the top 10 pathways as a table. DynOVis provides a function to download the complete list of genes and associated pathways.

## Results and discussion

As has been discussed in the introduction, there are multiple applications that aim to visualize biological networks. Here, DynOVis will be compared with the most popular tool at this moment: Cytoscape. Cytoscape is a stand-alone network visualization tool that offers multiple network analysis methods.

The workflow of DynOVis is designed in such a way from start to end, thus uploading a network file to visualizing a network, is straightforward to the user. Here, a comparison will be made between the workflow of DynOVis and Cytoscape. Both Cytoscape and DynOVis can handle different input formats, including .txt, .csv or .sif files. Whereas DynOVis has an automated feature to translate the two columns of input data (parent node and child node) to a network, Cytoscape first asks the user to specify the parent and child node column. This allows the user more freedom with respect to the number of columns in their input file, whereas for DynOVis the first and second column has to imply the network structure. Although the automated process restricts the user in their choices, it makes the visualization process more straightforward.

One of the most important features is the dynamic network visualization. DynOVis directly offers this feature, whereas for Cytoscape different apps need to be installed from their app store.

The Cytoscape app store contains multiple applications that allow for dynamic network visualization, such as DyNet [17], CyAnimator [11], DyNetViewer [18] and ANIMO [19]. ANIMO has been excluded from the comparison since the focus is more on signalling networks. In most cases, the user would like to study the perturbations of nodes over time or dose points in one biological network. Both DynOVis and CyAnimator have been built to show dynamic expression changes in nodes. DynOVis takes the dynamic input for every node and assigns these values as a colour to each node, while directly building the animation. For CyAnimator, the user has built each frame manually before the frame can be added to the animation. Each time point requires a step in which the node expression values have to be set as fill colour, which will be stored as a key frame. As a consequence, if there are ten different time points the user has to build ten key frames for each data point before the animation can be created. DynOVis saves the users some time because they only need to push the play button to start the animation.

Animations serve very well to identify important expression changes in the network, for instance, a gene that shows a strong downregulation between two time points. If these events are observed, the user would like to analyse the local network around this node. DynOVis makes this possible by highlighting a local neighbour network around a selected node and the user may play the animation or analyse the changes frame-by-frame. It is also possible to drag and drop the nodes to new places and start the animation. However, these two features are not possible while using CyAnimator. The node and edge positions are fixed after creating the animation and the animation is only visible in one orientation. If a local neighbour network around a node has to be analysed, the user has to create a new animation by hand.

Dynamic node interactions may be studied by using DynOVis, DyNetViewer or DyNet. DyNet is a Cytoscape app that visualizes differences among multiple networks, such as edges between nodes. This is of interest if one wants to study protein-protein-interaction networks in different tissues. The different node degree calculations can be performed to get the most important nodes between each network. DyNetViewer can generate a dynamic network by adding time course data to a static network. This will results in an animation with the different edges between nodes for the different time points. DynOVis can also generate an animation of the different changes in node interactions after the user uploads a file containing the interaction information per time point. The advantage of DynOVis over DyNet is that the latter requires at least two networks to compare, whereas DynOVis shows dynamic edge interactions in one network. DyNetViewer also shows dynamic edge interaction, but it calculates the interactions from the node expression values in a static network. This means that if one obtains a dynamic network with already known interaction changes, it cannot be uploaded in DyNetViewer. Here DynOVis has the advantage, because it can map dynamic edge interactions onto a network. DyNetViewer has an advantage if the dynamic edge interactions are yet unknown, because it calculates them from node expression data alone, something DynOVis cannot do from scratch. However, DyNetViewer cannot show the expression data on the nodes while playing the animation. DynOVis is capable of showing this information and therefore the user will see whether changes in node expression are actually altering node interactions.

With DynOVis we offer the implementation of dynamic network, functional and graph theory analysis without limitations with respect to the type of network. It is possible to study dynamic effects without the need for multiple networks while the tool immediately provides the user with information about gene function, associated pathways and diseases. Although Cytoscape is more powerful in analysing a static network, DynOVis has some advantages over the current Cytoscape applications regarding dynamic visualization (see Table 1 for overview). The combination of dynamic visualization and functional biological annotation is one of the advantages of DynOVis. Some of these functionalities are demonstrated in the following case studies.

**Table 1:** Overview of the different features present in DynOVis, Cytoscape and the different Cytoscape applications

Tools	DynOVis	Cytoscape	DyNet	CyAnimator	DyNetViewer
Platform	web	Standalone Java application	Cytoscape plugin	Cytoscape plugin	Cytoscape plugin
Input Network	List of interactions, Adjacency matrix, .sif file	List of interactions, Adjacency matrix, .sif file, URL or database	Cytoscape input types + 2 equal networks	Cytoscape input	Cytoscape input
Input data	Text file, CSV file, excel workbook	Text file, CSV file, Excel workbook, URL or database	Text file, CSV file, Excel workbook, URL or database	Text file, CSV file, Excel workbook, URL or database	Text file
Built-in database	Yes	No	No	No	No
Network layout	Force directed layout	Multiple different layouts	Cytoscape Layouts	Cytoscape Layouts	Cytoscape Layouts
Weighted edge visualization	No	Yes	Yes	Yes (from Cytoscape Core function)	No
Dynamic node expression	Yes	No	Yes	Yes	No
Dynamic node interactions	Yes	No	Yes	No	Yes
3D visualization	Yes	No	No	No	No
2D Line graph expression values	Yes	No	No	No	No
Pathway histogram	Yes	No	No	No	No

### Case studies on time series drug treatment experiments

Here we present two exemplar studies for the purpose of highlighting the importance of studying dynamic networks thereby indicating how DynOVis can aid in the analysis of the network.

**Fig. 2:**
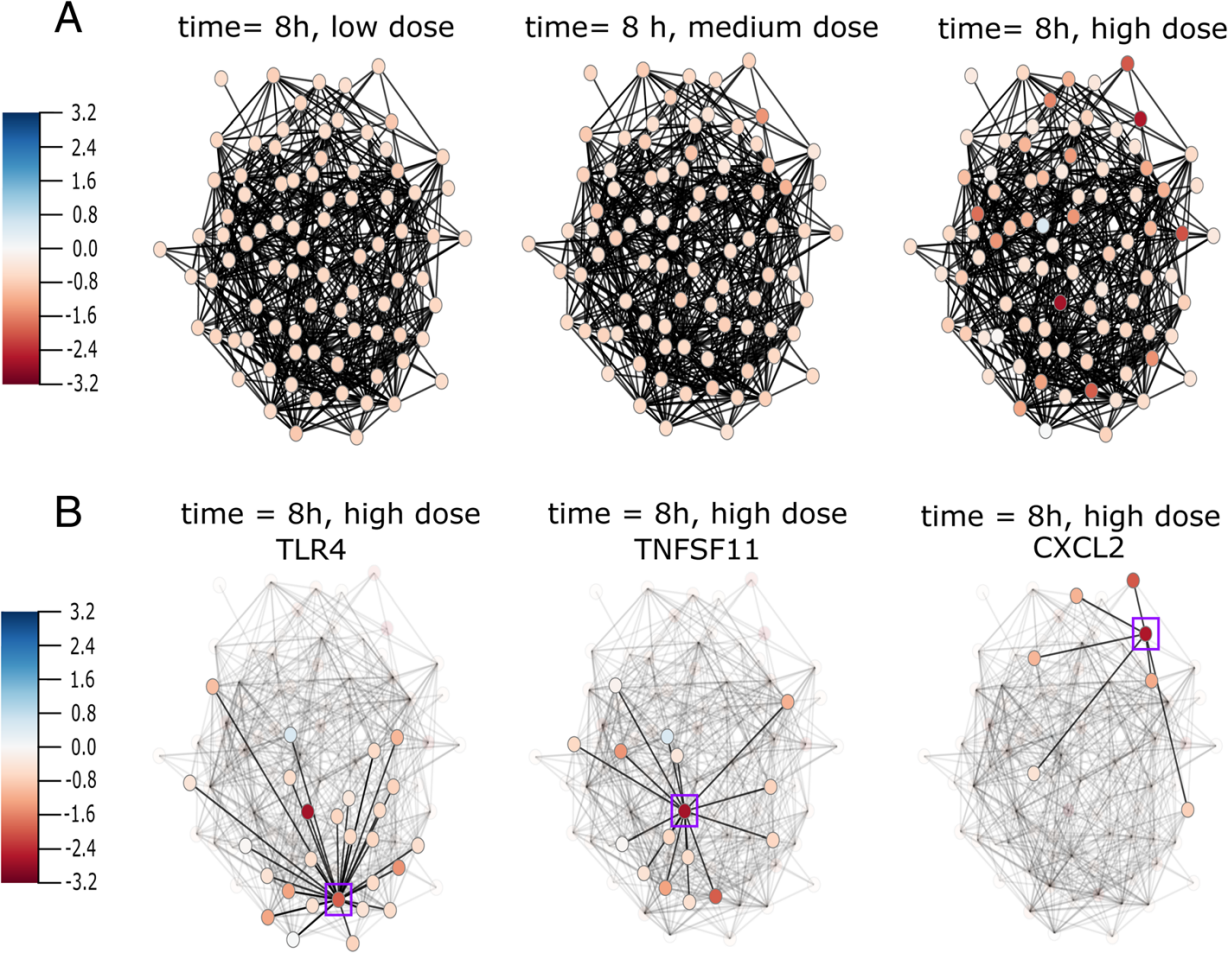
Frame-by-frame view of the expression profile in the NF-kB pathway at 8 h for low dose, medium dose and high dose of acetaminophen (Blue colour: upregulation, red colour: downregulation). After 8 h of exposure at high dose, a change in gene expression has been observed (panel **a**, low, medium and high dose compared). TNFSF11 and TLR4 show downregulation at 8 h (Panel **b**, node in purple square), whereas CXCL2 shows a strong downregulation at 24 h at high dose (Panel **c**, node in purple square)

In the first example, the network describing the NF-kB pathway has been investigated, a pathway related to apoptosis and inflammation, key events for both drug-induced liver fibrosis and cholestasis [20]. Here, a dose-over-time network of the NK-kB pathway was constructed by DTNI [8] from human in vitro samples exposed to acetaminophen from TG-GATEs [21]. Data were measured for two biological replicates at three doses (low, middle, high) at three time points (2, 8 and 24 h). By investigating the acetaminophen dose response of the NF-kB pathway over time, it becomes apparent that both time and dose play an important role in the changes in gene expression. From the animation it becomes clear that the induced effect of the acetaminophen challenge is dose- and time-specific, because the alternations in gene expression are only observed at a high dose after a period of 8 h (Fig. 2a).

TNF superfamily member 11 (TNFSF11) and toll like receptor 4 (TLR4) show a strong downregulation at 8 h (Fig. 2b), whereas C-X-C motif chemokine ligand 2 (CXCL2) shows a strong downregulation at 24 h (Fig. 2c).

**Fig. 3:**
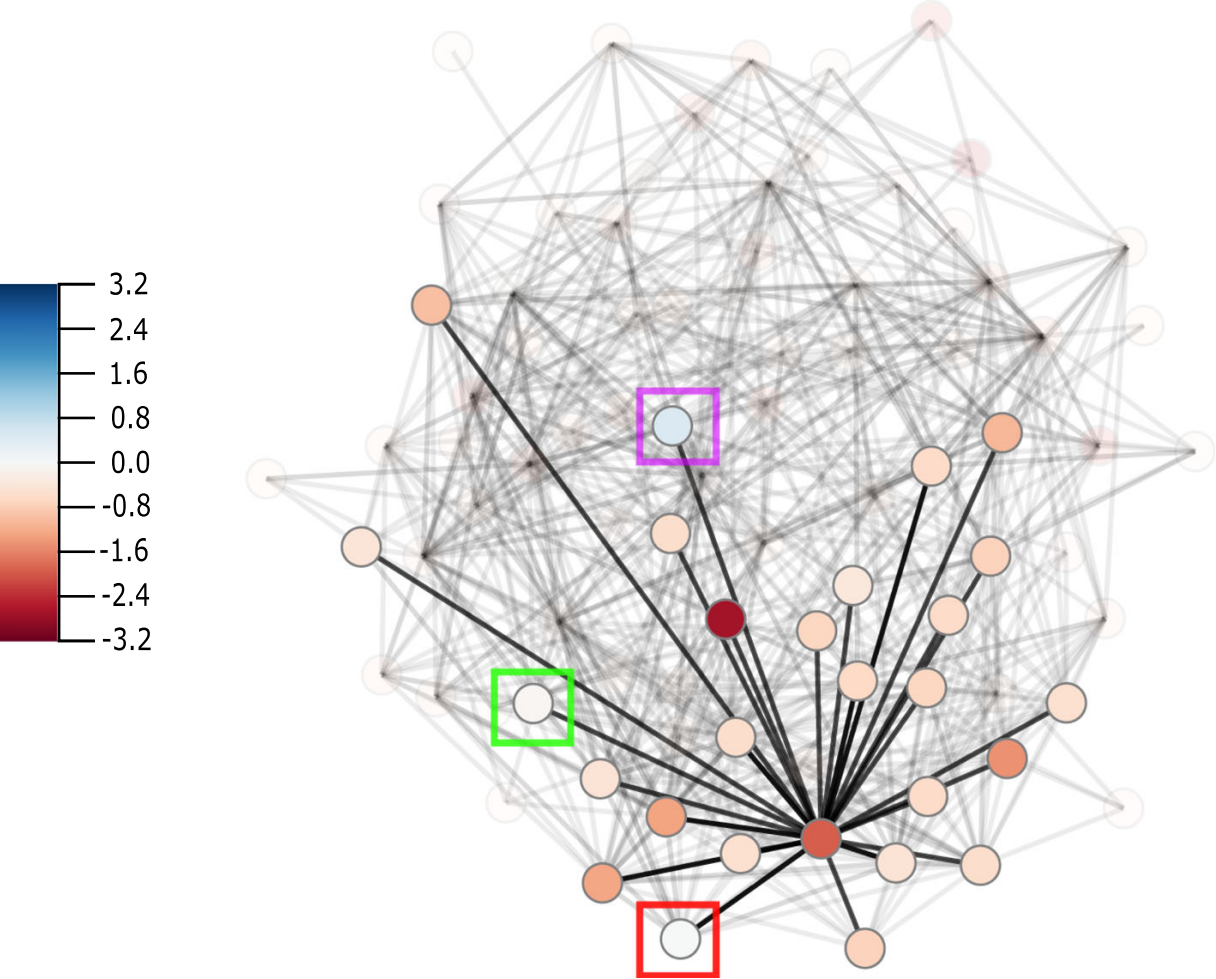
The highlighted selection of the first degree neighbours of TLR4 (central node in the sub selection) at time point of 8 h and high dose. CCL4 (green square), GADD45B (purple square), TNFSF13B (red square) show upregulation

**Fig. 4:**
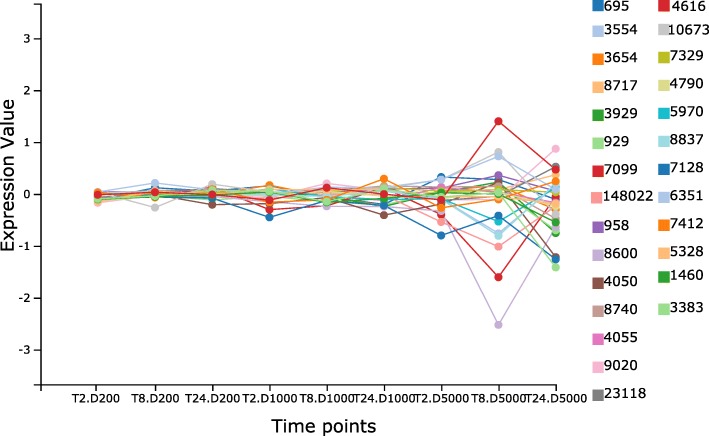
2D line graph of expression profiles of TLR4 and the first degree selected neighbours at different time and dose points. Time is abbreviated by T followed by 2, 8 or 24 h. Dose is abbreviated by D followed by 200 μM (low dose), 1000 μM (medium dose) and 5000 μM (high dose). Data is derived from human in vitro samples exposed to acetaminophen

TLR4 plays an important role in pathogen recognition and can activate the innate immune system [22]. It has been found in previous research that downregulation of TLR4 is related to liver cirrhosis [23]. Highlighting the neighbours of TLR4 using DynOVis (Fig. 3) demonstrates that a number of genes interacting with TLR4, show the same expression patterns and thus may play the same role in the response. TNFSF11 is one of those genes that has an interaction with TLR4 (downregulated at 8 h and high dose, dark red node in Fig. 3) and is involved in the regulation of the T cell-dependent immune response. While TLR4 shows a downregulation, three genes show an upregulation: C-C motif chemokine ligand 4 (CCL4), growth arrest and DNA damage inducible beta (GADD45B) and tumor necrosis factor ligand superfamily member 13b (TNFSF13B) (light blue nodes in Fig. 3, CCL4 green square, GADD45B purple square, TNFSF13B red square). GADD45B belongs to the GADD nuclear protein family that is associated with DNA Damage [24]. These results can also be saved in a 2D line graph, to show the changes in gene expression in a static way (Fig. 4).CXCL2 is one of the chemokines that leads to an influx of inflammatory cells including macrophages which are known for wound healing [25]. Downregulation of CXCL2 is observed at 24 h and therefore could play an important role in the toxic effect of acetaminophen on the liver. In this case study, DynOVis did help to highlight the most important genes in a high-density network as well as to identify important gene-gene interaction effects.

**Fig. 5:**
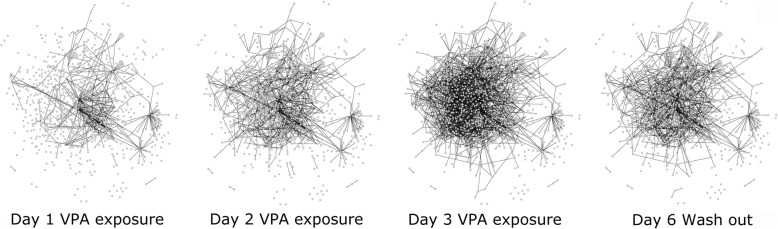
Dynamic molecular interaction changes over time after VPA exposure after 1, 2 and 3 days, as well as after the 3 day wash out period

In the second example, DynOVis is applied to a network with changing interactions over time as a response to valproic acid (VPA) exposure [26]. In this study, primary human hepatocytes are exposed to VPA for a period of 3 days with a follow up wash out (WO) period of 3 days. A selection of genes has been made by Wolters et al. [26] by applying the induced network module of ConsensusPathDB [12] to create multiple gene-gene interaction networks to unravel the dynamic changes over time after VPA exposure. From those results, only the high-confidence protein interactions and gene regulatory interactions are exported. We have used the edge interaction function of DynOVis to create an animation of the dynamic interaction changes over time that highlights important network structure changes (Fig. 5).

**Fig. 6:**
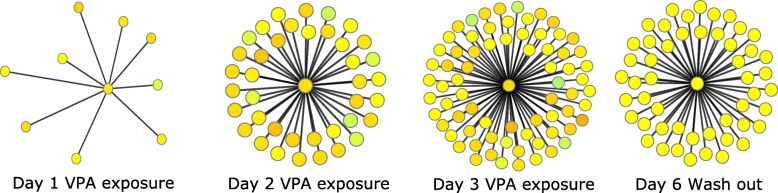
Subnetwork for Fibronectin 1 at the different days of VPA exposure. From this figure it can be seen that there is a strong time effect of VPA exposure, due to the increasing interactions over time. The centre node of each small subnetwork represents Fibronectin 1. At day 1 of exposure, there are 9 genes that have a gene-gene interaction with Fibronectin 1, this number increases over the following 2 days to 72 gene-gene interactions. The number of interactions decreases after the washout period

From Fig. 5, it becomes clear that the number of interactions grows over time during VPA exposure, with the highest number of interactions on day 3, which is in line with the observations of Wolters et al. [26]. A number of persistent gene-gene interactions are identified after the WO period. To fully understand the relationship between perturbations in gene expression and gene-gene interactions, DynOVis is used to create an animation that combines node expression with edge interactions. Here we will discuss only one gene of interest: Fibronectin 1 (FN1) (Fig. 6, network of FN1). FN1 is initially connected to 10 neighbours (outer degree: 3, inner degree: 7), but develops to 71 interactions (outer degree: 22, inner degree: 49) with other genes after a 3-day treatment with VPA and maintains in total 50 of the 71 interactions (outer degree: 15, inner degree: 35) after the 3 day washout period. The changes in gene-gene interactions between FN1 and its neighbours, as well as the changes in gene expression, are of great interest for various reasons. Knock-down of FN1 has been shown to play a role in mitochondrial-dependent apoptosis [27] as well as increased fibrosis in mice [28]. Accumulating cancer research showed that fibronectin expression in various tumours is highly correlated with malignant phenotypes and poor prognosis [29–31].

By identifying the dynamic interactions over time, we found both persistent and non-persistent interactions. The non-persistent interactions between FN1 with amyloid P component, serum (APCS) and filamin A (FLNA) could be associated with VPA exposure since the interactions disappear after the WO period. The expression pattern of APCS and FLNA are highly similar to FN1, which could indicate that the downregulation of those three genes plays a role in hepatotoxicity. A number of interactions disappear over a 2 day VPA exposure, including the interactions between FN1 and inter-alpha-trypsin inhibitor heavy chain 2 (ITIH2) and proliferating cell nuclear antigen (PCNA). These different dynamic gene-gene interactions are of interest because they may play a role in the same biological processes that lead to the adverse outcome of VPA exposure [32]. PCNA is a nuclear protein involved in DNA-synthesis and repair and decreased expression of PCNA has been experimentally shown in tumours of VPA treated mice [33]. ITIH2 belongs to the family of plasma serine protease inhibitors involved in stabilization and prevention of tumour metastasis. Further investigation of the relationship between these genes is needed but is behind the scope of this case study. In the second case study, DynOVis gives us a quick overview of the growing gene-gene interactions over VPA exposure, which helps to identify important time points in the exposure series and directs us towards the most important genes to study.

## Conclusion

With DynOVis we offer the implementation of dynamic network visualization, by providing the users with functionalities to highlight node expression changes and dynamic edges. The addition of biological information, such as pathway or disease association, helps to further understand the role of different nodes in the network. It is possible to study dynamic effects without the need for multiple networks while the tool immediately provides the user with information about gene function, associated pathways and diseases. Although Cytoscape is more powerful in analysing a static network, DynOVis has some advantages over the current Cytoscape applications regarding dynamic visualization. DynOVis allows studying both dynamic node expression changes and edge interaction changes simultaneously, whereas the current Cytoscape tools focus more on one topic. With the provided case studies, we have shown that with the dynamic network visualization it becomes less complicated to identify important causal events. Further development of the tool will be carried out, in order to enable the integration of multiple omics platforms that will provide an even more detailed explanation of the cellular response to perturbations. These updates will be integrated into the tool and updated regularly.

## Availability

**Project name:** DynOVis.

**Project home page**: https://tjmkuijpers.shinyapps.io/dynovistool/ and https://bitbucket.org/mutgx/dynovis/src/master for source code.

**Operating system(s):** Operating system independent (web service).

**Programming Language:** R and JavaScript.

**Other requirements:** The DynOVis interface uses HTML5 features that are not supported by the current version of Internet Explorer (IE v11); i.e. Internet Explorer v11 does not fully support DynOVis. Therefore, we recommend using DynOVis on a different browser (Google Chrome or Mozilla Firefox).

**License:** BSD license.

**Any restrictions to use by non-academics:** license needed.

## Data Availability

Source code and the data used for the two case studies are all available through https://bitbucket.org/mutgx/dynovis/src/master/
